# Reward monitoring in the frontopolar cortex of macaques

**DOI:** 10.1038/s41598-025-99019-3

**Published:** 2025-05-12

**Authors:** Lorenzo Ferrucci, Francesco Ceccarelli, Fabrizio Londei, Giulia Arena, Leyla Elyasizad, Simon Nougaret, Aldo Genovesio

**Affiliations:** 1 Department of Physiology and Pharmacology, Sapienza University of Rome, Piazzale Aldo Moro 5, 00185 Rome, Italy; 2 Institute of Biochemistry and Cell Biology (IBBC), National Research Council of Italy (CNR), Via Ramarini 32, 00015 Monterotondo Scalo, Rome, Italy; 3 Behavioral Neuroscience PhD Program, Sapienza University, Rome, Italy; 4 Institut de Neurosciences de La Timone, UMR 7289, Centre National de La Recherche Scientifique and Aix-Marseille Universite, Marseille, France; 5 Department of Pharmaceutical Sciences, University of Piemonte Orientale, Novara, Italy

**Keywords:** Frontal pole, Area 10, Monkey, Reward, Explore-exploit, Learning, Reward, Cortex, Neurophysiology, Problem solving

## Abstract

Reward processing involves several prefrontal cortex areas, enabling individuals to learn from behavioral outcomes and shape decisions. However, the role of the frontopolar cortex (FPC) in these processes remains unclear due to limited single-neuron research. In this study, we recorded neural activity from the FPC of two macaques performing a fast-learning task, the object-in-place reward task, which examined how reward size affects learning. Results showed that FPC feedback monitoring activity extends to the value of specific choices. Moreover, once the association between scenes and reward had been learned, FPC neural activity before choice reflected the future animal’s behavior to stay or to switch on their previous behavioral strategy, i.e., to choose the same target or the other one. These results suggest that FPC neurons integrated information for action monitoring and later reprocessed it to decide the best behavioral strategy to adopt, determining whether to maintain or change the action plan.

## Introduction

The frontopolar cortex (FPC) or frontal pole is a brain region that developed only in anthropoid primates, reaching its greatest extension in humans. For this reason, its functions have been linked with the highest cognitive functions specific to primates^1–3^. It has been hypothesized that it plays a critical role in processing stimulus independent thought^4–6^ and in differentiating between externally and internally driven information^7^, contributing to the building of self-referential knowledge. Other studies suggested that its main functions involve mnemonic processes, more precisely in information retrieval in memory^8,9^, perspective memory^10–12^ or in holding in mind goals while performing other actions or exploring alternative goals, as described by the theory of cognitive branching^13^. Indeed, it has been found to be implicated in humans in exploratory behavior^14–16^, signaling uncertainty regarding available alternatives^17,18^. This suggests that this area supports exploration-based decision-making by keeping track of alternatives and by redirecting attention to available but unchosen options^19–21^, with a parallel processing that allows the extraction of multiple information from the surrounding environment^22^. In addition, lesion studies showed that the extent of frontopolar lesions in patients can predict their impairment in multi-tasking^23^, and that lesions of the most anterior part of the prefrontal cortex affected more tasks which require to implement abstract action rules compared to concrete action rules^24^. In parallel, lesion studies in monkeys have shown that FPC is necessary for exploration, fast learning^25^ and to control and adapt response execution^26^. In addition to the results mentioned above, studies examining the behavioral consequences of FPC lesions have largely shown no significant effects on most cognitive processes^27^. This has led to the hypothesis that the FPC’s primary role lies in coordinating information processing across various prefrontal cortex regions, rather than directly contributing to the execution of specific cognitive tasks^28^.

Compared to other prefrontal cortex areas, the main theories on FPC functions emerged from neuropsychological and fMRI human studies previously mentioned, since FPC has been less investigated in neurophysiological studies, mainly due to the difficulty to surgically implanting electrodes in this structure for neural recordings in non-human primates^29^. From macaques’ electrophysiological recordings, it has mainly emerged that FPC neurons are involved in monitoring one’s own and others’ actions when the choice is made^30–33^, encoding the position of the chosen target. Recently, it has been reported that FPC neurons could also respond during a delay period, but only in defined contexts, when monkeys were involved in a fast-learning task^34^. Once the stimulus-action-outcome association was learned, the representation of the goal position appeared before the choice was made, together with an overall higher activity during the exploration of new scenes.

**Fig. 1:**
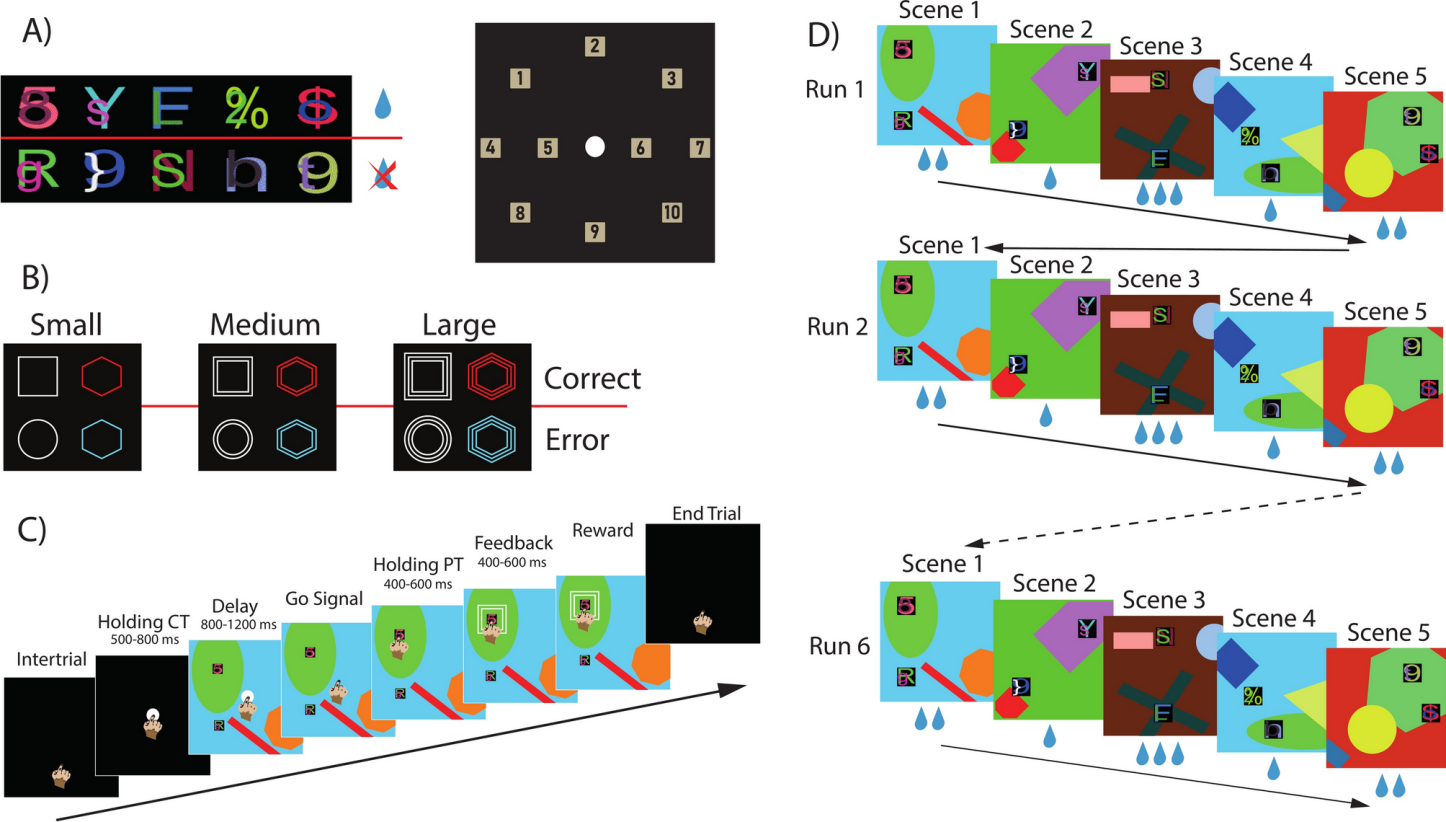
Object in Place Reward (OIPR) task and trial’s structure. (**A**) Examples of different objects used for a specific block (left) and the ten possible locations where objects could be presented in pairs on the screen (right). New stimuli never seen before by monkeys were created for each new block. (**B**) Visual stimuli used as feedback to indicate correctness and reward size. (**C**) Temporal sequence of a trial. (**D**) Temporal sequence of a block. The number of drops below each scene illustrates the reward size associated with that specific scene that was kept constant among runs.

How can a bridge be found between the numerous hypotheses about the functioning of the FPC derived from the human studies’ literature and the results obtained in electrophysiology on non-human primates? The key aspect that emerges from the sparse neurophysiological literature about the properties of FPC neurons is that they are involved in monitoring the spatial position of a chosen target, and that this information is subsequently recalled before the choice is made once learning is completed. In the current study we asked whether this monitoring activity could be a general feature of the FPC, extending also to other features, such as the value of a performed action. To investigate this hypothesis, we have adopted a variant of the object-in-place task^25,35,36^ used in our previous behavioral study^37^, the object-in-place reward task (OIPR), during which we recorded the neural activity of FPC neurons from two rhesus macaque monkeys. The monkeys were presented with five scenes with two targets in two different possible spatial locations (Fig. 1A). The choice of one target led to the receipt of a liquid reward, while the other did not. Unlike the previous version, however, where the amount of reward was fixed, in this version each scene was associated with a different reward size: small, medium, or large. The size was signaled by a visual feedback following the choice (Fig. 1B). Each scene was presented on the screen for a fixed amount of time before the monkeys were allowed to choose one of the two targets and receive the feedback (Fig. 1C). When the five scenes were presented, the first run was concluded, and the same scenes in the same order were presented again for a total amount of 6 runs (Fig. 1D), offering the monkeys the opportunity to exploit the information learned during the first run and gain further rewards. The OIP task is designed specifically to study fast learning. The learning rate is accelerated thanks to the redundancy of information present in each scene: the presence of only two targets with unique features, specific spatial locations, and the presence of an ever-changing background ensure the uniqueness of the scene as a whole. This kind of fast learning seems to be mediated by the FPC, being impaired in monkeys with lesions of this area^25^, suggesting that it is supported by the learning of the value of the two alternative targets. For these reasons, we chose the OIPR task to investigate a possible neural correlate in the FPC of different reward sizes representing the value of a chosen target. In a previous behavioral study using the same task, we showed that the learning rate was influenced by the reward context, i.e. the different amounts of reward available in the task and their relative differences, rather than its absolute value^37^. Here, we found that learning the association between the scene and the correct target was influenced by the amount of reward. At the neural level, reward size was represented in two different modes depending on the task epochs. At the feedback presentation, we found a clear representation of the 3 reward sizes informing the monkeys about the outcome of their actions. Interestingly, we did find responses during the Delay epoch. When monkeys made their choice, the neural representation of the medium and large reward were similar, while that of the small reward was different. This signal might drive the animal’s decision to choose the same object in medium/large reward conditions or switch toward the alternative object in the small reward condition.

## Results

### Learning is influenced by reward size

**Fig. 2:**
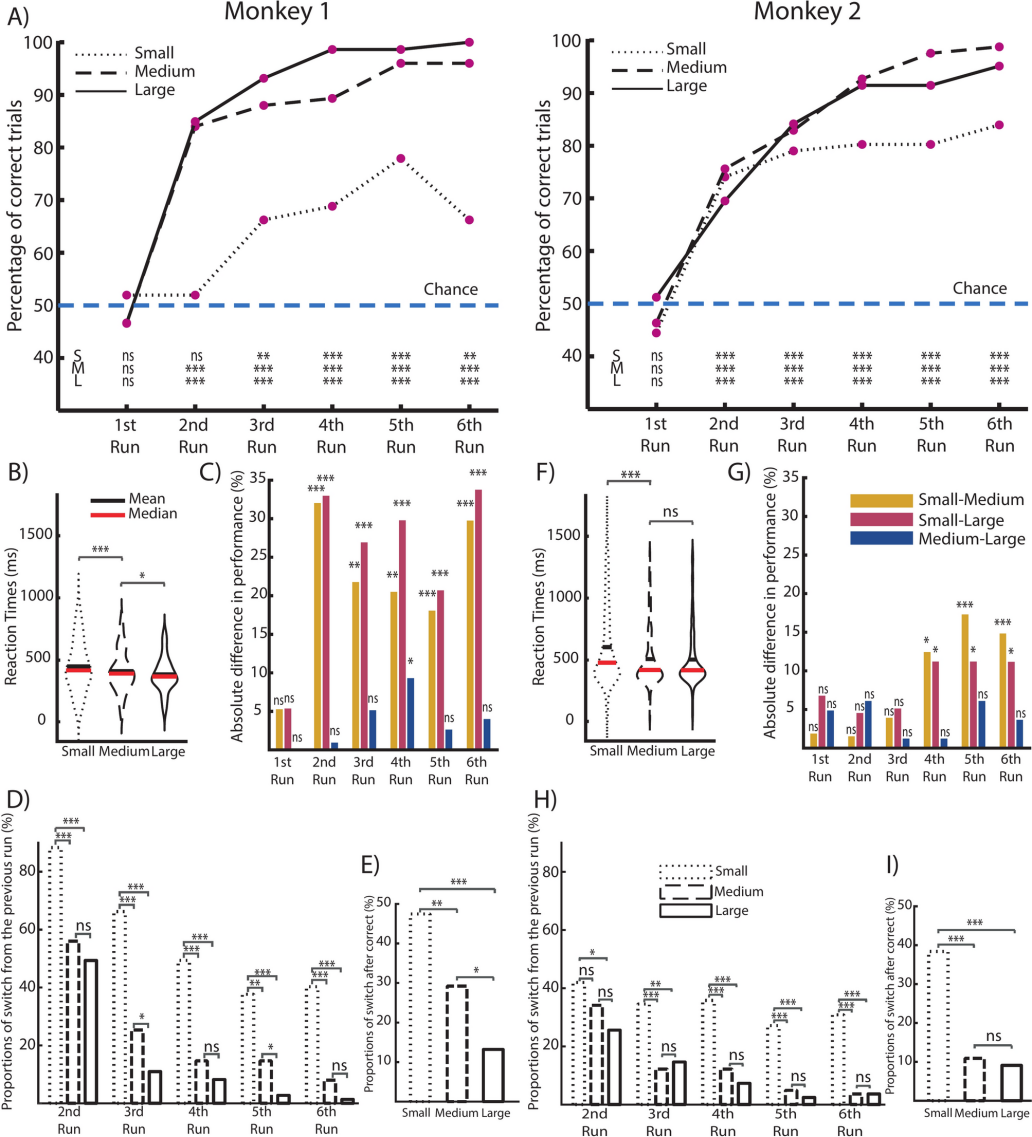
Behavioral results. (**A**) Percentage of correct trials for each run divided by reward size, for Monkey 1 and Monkey 2 separately. Difference from chance is indicated at the bottom of the plot for every run and every reward size (S, small; M, medium; L, large reward size, respectively). ns: not significant; *: p < 0.05; ** p < 0.01; ***: p < 0.001, binomial test. Blue dashed horizontal line represents the chance level of performance. (**B**,**F**) Violin plot of reaction times for trials of each reward size, for Monkey 1 (**B**) and Monkey 2 (**F**). ns: not significant; *: p < 0.05; ** p < 0.01; ***: p < 0.001, two-sample Kolmogorov Smirnov test. (**C**,**G**) Absolute difference in performance between reward sizes for each run, for Monkey 1 (**C**) and Monkey 2 (**G**) separately. ns: not significant; *: p < 0.05; ** p < 0.01; ***: p < 0.001, Chi-squared test. (**D**,**H**) Proportions of switch trials, for each run (Run 1 excluded) and for each reward size, for Monkey 1 (**D**) and Monkey 2 (**H**) separately. Switch trials are defined as those scenes in which the monkey chose a different target than the one chosen in the same scene at the previous run. ns: not significant; *: p < 0.05; ** p < 0.01; ***: p < 0.001, Chi-squared test. (**E**,**I**) Proportions of switch trials after a correct trial for each reward size for Monkey 1 (**E**) and Monkey 2 (**I**). ns: not significant; *: p < 0.05; ** p < 0.01; ***: p < 0.001, Chi-squared test.

We calculated the percentage of correct trials for each run and for each reward size for both monkeys (Fig. 2A). Monkey 1 already had experience with the task prior to the start of recording. During Run 1, monkeys were unfamiliar with the scene, and the only possible strategy to adopt was a guessing strategy, exploring one of the two targets and leading performances at chance in every condition. From the second run, both monkeys performed above chance in medium and large reward conditions. They quickly reached and maintained until the 6th run a high percentage of correct trials. In contrast, in the small reward condition, Monkey 1 showed a slower increase in performance compared to the other two conditions, with a percentage of correct trials significantly higher than chance only from Run 3 and still significantly lower than those in the other two conditions (Fig. 2A, left). For Monkey 2, the performance in the small reward condition was above chance from Run 2, where it reached a maximum without increasing and remained significantly lower than in the other two conditions from Run 4 (Fig. 2A, right). Analysis of reaction times (i.e., from go-signal to the onset of movement toward the chosen target) showed significantly longer times in both monkeys in trials of the small reward condition than in the medium and large reward conditions (Fig. 2B,F). The difference in performance between the different reward sizes was only significant when comparing the small reward condition with the other two from Run 2 onwards for Monkey 1 (Fig. 2C), and from Run 4 onwards for Monkey 2 (Fig. 2G), while the difference in performance between the medium and the large reward conditions showed no significant difference in either monkey in any run (with the exception of a small difference in Run 4 for Monkey 1). In a complementary manner, the high level of performance attained in the second half of the block (3 to 6 runs) in the large and medium reward conditions can be interpreted as a higher capacity to exploit the information about the correct target learned in the first part of the block, consistently repeating the same choice, as opposed to the small reward condition, where a more exploratory type of behavior is enacted, which slows down the learning curve. To test this, we calculated for each run and reward size the proportion of trials (i.e., scenes) in which the monkeys chose a different target than the one chosen in the same scene at the previous run (switch trials, Fig. 2D,H). Both monkeys showed a higher proportion of switch trials in the small reward condition in all the runs, while medium and large reward did not show any substantial differences. The tendency to explore the alternative target and then make a switch trial in the small reward occurs regardless of whether the previous trial is correct or incorrect in both monkeys (Fig. 2E,I), whereas this is not the case in the medium and large reward trials. In addition, we analysed the eye data of monkey 2 to investigate, during the Delay epoch, the percentage of time spent observing the correct target, the wrong target and the central target across runs. The results, as already shown in a previous work using the same task design^34^, show a tendency to explore the two targets less across runs, while the central target is observed for a longer time across runs (Supp. Figure 1, top). We then repeated the same analysis of the eye data but divided by reward size instead of run. The results show that in the small reward condition during the Delay epoch, Monkey 2 explored the correct and wrong targets equally. In contrast, for the medium and large reward conditions, there was less target exploration, and the most observed target was the correct one (Supp. Figure 1, bottom). As a further check, we repeated the analysis of the behavioral data by averaging the values computed for each recording day per animal (4 days for Monkey 1 and 10 days for Monkey 2).The behavioral results followed a similar pattern using both methods (Supp. Figure 2). The complementary proportion of switch trials after incorrect trials is shown in Supp. Figure 3.

### Reward size is encoded at feedback and during the presentation of the scene when learning occurs

**Fig. 3:**
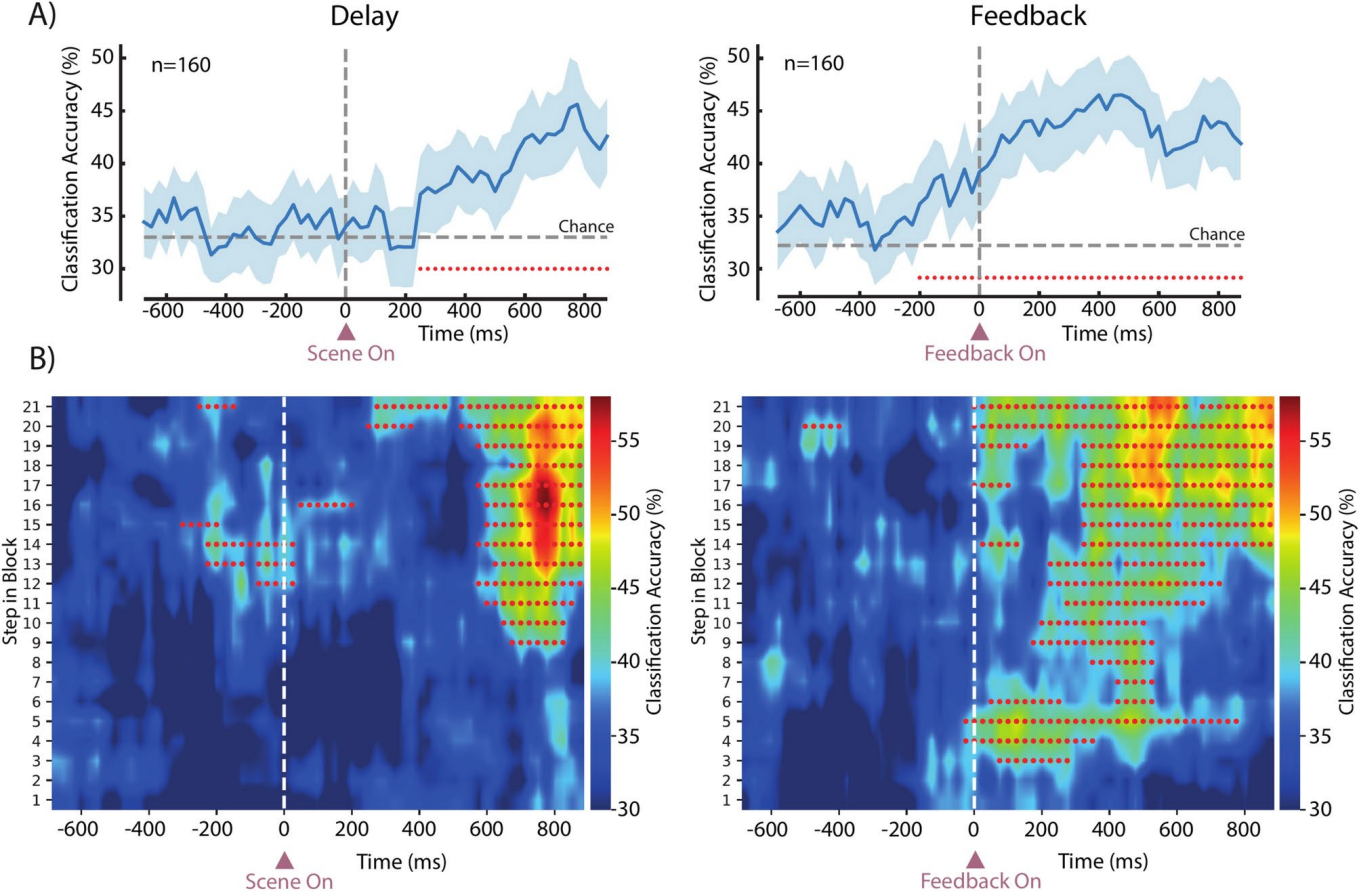
Decoding reward size from population activity. (**A**) Classification accuracy for reward size (small vs medium vs large reward trials) over time in the two epochs of interest: Delay and Feedback. Shaded areas represent standard deviation over different resamples. Red dots indicate a series of at least five consecutive bins with classification accuracy significantly higher than chance. Dashed horizontal line represents the chance level of decoding accuracy. (**B**) Classification accuracy for reward size in the population of neurons during the two epochs as a function of the number of trials in a block. Red dots indicate a series of at least five consecutive bins in a specific step with classification accuracy significantly higher than chance. Color bars show classification accuracy values.

We focused our analysis on two key epochs of the task, namely the Delay epoch (after scene presentation and before go-signal) and the Feedback epoch (i.e., after presentation of the visual stimulus that indicated reward size but before actual reward delivery). We predicted two different activity patterns in these epochs based on the literature on reward-related processes in cortical and subcortical structures. During the Feedback epoch, the information represented should reflect the received outcome and be sent to other areas to inform about the output of performed action; this monitoring activity is essential to learn the specific associations. Thus, we should observe a clear distinction between the three reward sizes. Differently, during the Delay epoch, the information should represent the monkey’s behavior, i.e. how the monkey uses its knowledge related to the reward size associated with actions previously performed to make its future choice. In that case, we should observe a representation of the reward reflecting the monkey’s behavior, small reward vs medium and large reward conditions. To test this hypothesis, we trained and tested a SVM classifier to discriminate reward conditions within the population activity, using all completed trials regardless of the run. We found that information about the reward size was encoded both during the Delay (Fig. 3A, left), specifically in the later part after the presentation of the scene (400–800 ms after), and in the Feedback epoch (Fig. 3A, right), with the latter epoch that showed a higher accuracy compared to the former. Similar results were found for each monkey separately (Supp. Figure 4). As a control, we examined whether the neural response evoked by feedback presentation was influenced by the visual properties of the feedback stimuli (color and shape). We found that neurons in the FPC did not encode the shape or color of the feedback within the window between feedback onset and 400 ms afterward, in either correct (5 out of 160 neurons, 3.1%, one-way ANOVA, p < 0.05) or incorrect trials (4 out of 160 neurons, 2.5%, one-way ANOVA, p < 0.05). Since the reaction times were significantly influenced by the reward size (Fig. 2B,F), we performed an additional control analysis, aiming to disentangle the possible confound between motor and reward size modulations of the neural activity. For each neuron, we performed a linear model analysis, taking as regressors, the reaction-movement time and the reward size and their interaction. We then applied an ANOVA on the model and extracted the percentage of variance explained by each variable for each neuron. During the Delay epoch, among the neurons from which the full model provided a better fit than null (23/160, 14%), we observed that the variance explained by the reward size was higher than the variance explained by the reaction-movement time in the majority of them (16/23, 70%). Similarly, during the Feedback epoch, on the neurons from which the full model provided a better fit than null (33/160, 21%), we observed that the variance explained by the reward size was higher than the variance explained by the reaction-movement time in the majority of them (25/33, 76%). The number of significant neurons for each parameter is detailed in Supp. Table 1.

The information about the reward size was always present in the Feedback epoch, in contrast to the Delay epoch, where no cues were present in the scene indicating which reward size the scene was associated with, and the association scene-reward size could only be inferred after learning across many repetitions of a scene. For this reason, we further investigated how the representation of reward size evolved during the task, from the beginning to the end of a block. We applied the same decoding procedure within the same neural population but selecting steps of trials within each block (each step was the average of 10 trials, see Methods for details), to visualize how classification accuracy evolved in time during the six runs of the task. As expected, the encoding of reward size during the Delay epoch developed exclusively in the second part of each block (Fig. 3B, left), when the association between a specific scene and its relative reward size had been experienced several times and thus learned. In contrast, reward size encoding during the Feedback epoch appeared to be more homogeneous within a block (Fig. 3B, right), due to the presence of the visual feedback during this epoch, although again it strengthened as learning advanced.

### Similarities between neural representations of reward size

**Fig. 4:**
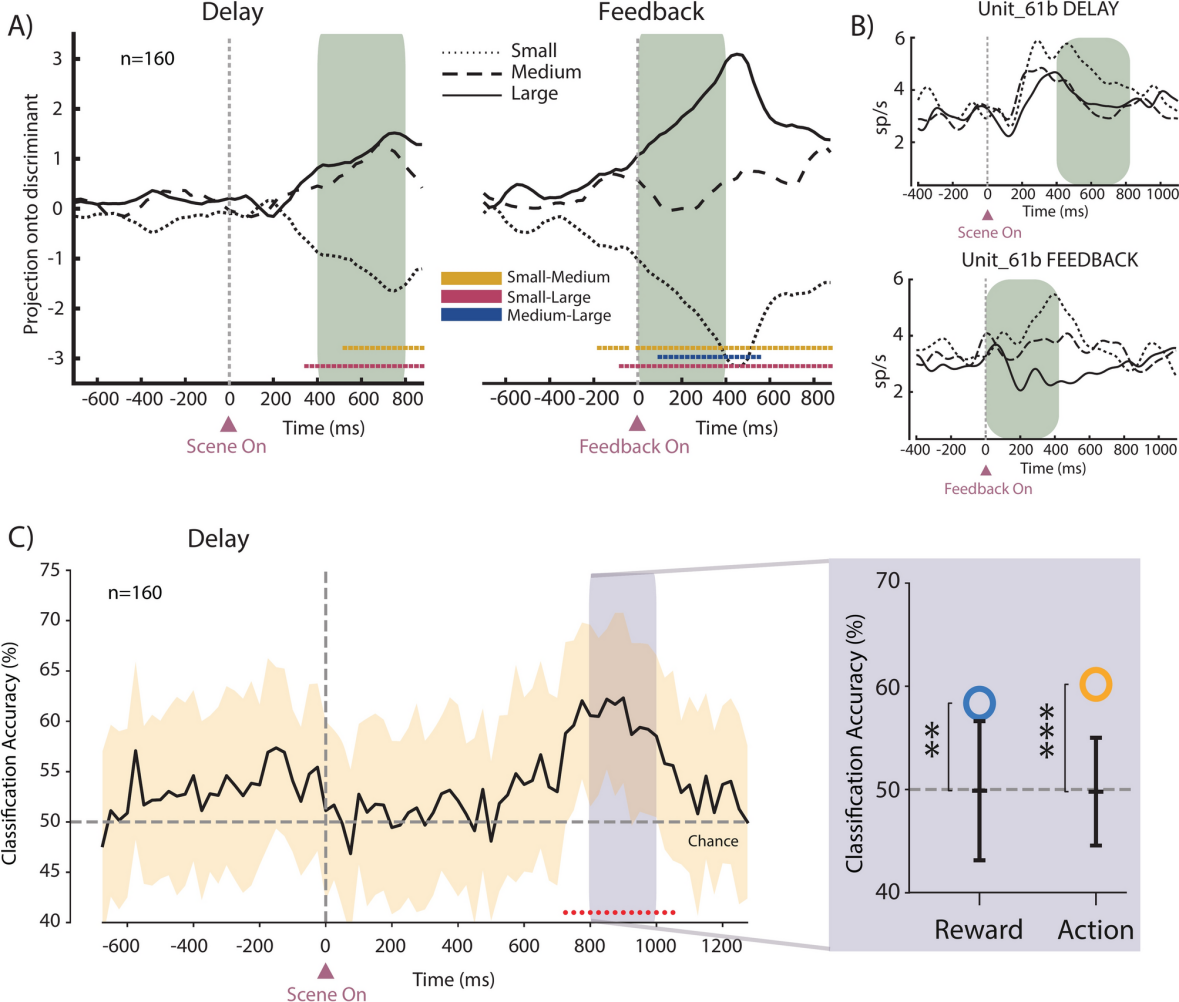
Similarities between neural trajectories over time. (**A**) Projections into the discriminant for each reward size during Delay (left) and Feedback (right) epochs. Colored dots indicate a series of at least five consecutive bins in which the values of pairs of projections differ from each other (overlap index < 5%). (**B**) Example of a neuron showing the same trend observed in the population trajectories, with a similar average firing rate between medium and large reward size during the Delay (top) and a different average firing rate between the three reward sizes during Feedback (bottom). (**C**) Classification accuracy for the action (stay vs switch trials) over time in the Delay epoch. Shaded areas represent standard deviation over different resamples. Red dots indicate a series of at least five consecutive bins with classification accuracy significantly higher than chance. Dashed horizontal line represents the chance level of decoding accuracy. In the purple box is shown the classification accuracy for both reward (blue circle) and action (yellow circle) in the selected 200 ms bin after the balanced sampling procedure to disentangle the variables from each other. Note that chance level is at 50% for both variables since medium and large reward trials were grouped together into a single label. ns: not significant; *: p < 0.05; **: p < 0.01; ***: p < 0.001.

Behaviorally, medium and large reward conditions were similar and both different from the small reward condition. We hypothesized that a neural representation of this behavior might reflect the motivation required for the animal to persist with the same behavioral choice in the medium and large reward conditions or to switch to the alternative target in the small reward condition. To test this hypothesis, we investigated the similarities across the evolution of neural trajectories in the two epochs of interest for the three reward conditions. A separated representation of the 3 reward sizes was expected at feedback occurrence to monitor the outcomes, whereas an analogous representation was expected during the Delay epoch to drive the animal’s choice. We represented the population activity in a N-dimensional space (where N is the number of neurons, 160 to match the same population used for decoding analysis) and examined whether the neural trajectories of different reward sizes converged or diverged onto a specific point in the state space. Neural trajectories of different reward sizes were indeed highly different depending on the epoch analyzed. As hypothesized, in the Delay epoch, during scene exploration, the projection of the medium reward size was not significantly different from that of the large reward size, while both were significantly different from the projection of the small reward size (Fig. 4A, left). This result shows a tendency to have the neural activity clustered into two subgroups rather than three, where medium and large reward sizes are similarly represented. In contrast during the Feedback epoch, the projections of the three reward sizes were different from each other, with that of the medium reward size lying equidistant between the small and large projections (Fig. 4A, right). A similar pattern was represented by the discharge activity of individual neurons encoding reward size distinguishing between the three classes at Feedback but with a similar firing rate for the medium and large reward at Delay (example in Fig. 4B). Similar results were found for each monkey separately (Supp. Figure 5).

Following our hypothesis, the separation of neural trajectories between medium/large and small reward conditions at the Delay could reflect an exploit/explore signal. We directly tested this hypothesis and investigated whether the action performed, i.e. stay/switch trials, was discriminated within the population activity. We trained and tested a SVM classifier to discriminate between these two categories of trials according to the same criterion used for the behavioral analyses (Fig. 2D,H). We found that the classification accuracy was above chance in a time window between the end of the Delay and the go-signal, approximately 200 ms after the period when the accuracy for reward size was significant (Fig. 4C), indicating a modulation for the stay-switch behavior. However, considering the animal’s behavior, there is a strong overlap between the trials labels when sorted by reward size (grouping together medium and large reward trials) and when sorted by action (stay or switch). To overcome this issue, we repeated the decoding in the 200 ms bin where we found the highest classification accuracy for the action, using a procedure designed to balance the variables and eliminate the confounding effects of correlation between them. The results show that both variables are found to be significantly encoded (Fig. 4C, grey box) independently of each other.

## Discussion

Our study tested whether the monitoring activity of FPC neurons was generalizable and extended to other features, such as reward value. Our results confirm that the representation of the reward magnitude was present in the FPC at the Feedback epoch since the beginning of the block and was maintained stably throughout the learning block. Population neural activity in the state space showed three distinct representations of reward size, divided into the three categories indicated by the visual feedback small, medium, and large. The coding of the reward size at Delay, on the other hand, exhibited different characteristics. Once the association between a specific scene and the reward size attached to it was learned, the reward size coding emerged also during the Delay epoch, i.e., at the time when the scene was represented in the runs following the first one. In contrast to the Feedback epoch, medium and large rewards were similarly represented in the state space, while small reward had a different representation.

These results refine the interpretation of previous findings and allow drawing new hypotheses on FPC functions in cognition. So far, FPC neurophysiological studies in macaques have shown an involvement of these neurons in monitoring actions performed or observed, encoding the spatial location of a target exclusively at the time of choice, except in a learning context where the monitored information is also recalled during a delay period preceding the choice. Our results suggest that the monitoring process can extend to different aspects of the choice, not limited to its spatial position during social and non-social tasks, as shown before^30,33,34^, but also to its value. FPC neurons encoded the reward size associated with each specific scene at the Feedback epoch, clearly discriminating between the three different reward sizes. This discriminative ability was related to the association between the stimulus, the action, and the size of the reward signaled by the feedback following them. This signal could serve as a report on the consequences of the performed action, here the amount of received reward, or, in other words, the value of a performed action, and has been described in cortical and subcortical structures^38^. Beside their role in the encoding of rules and strategies^39–42^, subjective decisions and economic choices^43–45^, orbitofrontal cortex neurons track information about the outcomes that follow actions, encoding the identity, the magnitude and the value of rewards^46–50^. Reward-related neural signals have also been found in different areas of the medial prefrontal cortex within a social context, where individual neurons are modulated by the amount or probability of a reward taken by another agent^51,52^. Similarly, several subcortical structures, which are connected with these cortical areas^53–57^, are thought to be involved in motivational processes whose neural basis rests on the ability to encode reward-related signals. Neurons in the hypothalamus and in the amygdala signals reward value both for self and others^58–60^, and it has been suggested that by encoding object values learned through observation they can build a representation that serves to construct a representation of others’ mental states^61^. Different structures of the basal ganglia are thought to be involved in reinforcement learning, through connection with dopaminergic neurons in the midbrain that encode reward expectation and reward prediction error^62,63^, or in general in decision-making processes based on the value of a reward^64–67^.

This large literature shows that the encoding of reward value has been found in several areas of the brain, thus it is not an exclusive of the FPC; similarly, monitoring the position of a chosen target, as seen in our previous studies^33,34^, is not an exclusive of the FPC. This raises the difficult question of what makes the FPC unique in comparison to the rest of the prefrontal cortex. However, the FPC may not have evolved to handle specific cognitive tasks. As suggested by previous studies^28^, FPC could work as a hub for these signals through its generalized ability to monitor relevant aspects of the performed action. It could act as a ‘monitoring center’, simultaneously integrating the outcomes of different cognitive processes occurring in different brain areas. This hypothesis is supported by the distinctive anatomical interconnections between FPC and the majority of prefrontal areas^68–72^ and is also in line with the virtual absence of impairment after FPC lesions^27^. Indeed, such lesions would therefore not affect the ability to perform basic cognitive tasks, which can be handled by the activation of other brain areas individually, but rather the ability to integrate different functions, which determines the efficiency with which certain cognitive processes are performed, a characteristic particularly evolved in anthropoid primates.

If FPC works as a monitoring center of multiple information, it remains however unclear whether and how this information is ultimately stored, eventually reprocessed and to what end. Our results show an encoding of the reward size also during the Delay epoch, before the action was performed, when the scene was subsequently represented in the next runs. This suggests that the features of an action, monitored at the time it was performed, can be recalled for later reuse at least in some specific contexts. Similarly, previous studies had found that spatial information in the FPC was recalled in the Delay epoch^34^, but only during a fast-learning task and not in a task where it was not necessary to keep the information in memory in subsequent trials^33^.

In addition, we found that the coding of reward size differed between Feedback epoch and Delay epoch, the three magnitudes were represented differently during these periods at the population level. During the Feedback epoch, population neural activity in the state space showed three distinct representations of reward size, divided into the three categories, small, medium, and large. When the scene was subsequently presented in the next runs, medium and large rewards were represented similarly, with both differing from small reward. This suggests that when information was recalled during the scene presentation, in runs following the first, it was no longer processed in terms of absolute size. It was indeed representing an integrated dichotomic signal distinguishing action-reward associations with high vs. low subjective values. This interpretation is supported by the similarity between the neural representation of the reward size in the Delay epoch and the learning curves divided by reward size observed in both monkeys. Indeed, scenes associated with medium and large reward sizes were learned faster by showing two learning curves that did not differ significantly from each other, while scenes associated with the small reward were learnt more slowly.

This interpretation can be taken further as FPC has recently been identified as a crucial area for explore-exploit decision-making^3,73–79^, guiding other areas of the prefrontal cortex in the decision on whether to pursue an ongoing behavioral strategy that has proved to be effective (exploitation) or interrupting it to switch toward a different alternative (exploration). To do this, the FPC is well positioned at the top of a hierarchical scale comprising a network of different prefrontal areas that support different cognitive functions^80–88^, providing FPC with task-related information of different nature, based on environmental cues and past experiences. This information would then be used by the FPC to orchestrate the best behavioral strategy to adopt through top-down connections on other prefrontal areas. Such a mechanism is critical not only for planning self-generated choices but also potentially for planning one’s own choices based on the outcome of socially observed ones, considering the prefrontal cortex’s ability to represent the value of others’ choices in a social context^89–92^. A recent study^93^ advanced the hypothesis that reward plays a crucial role in allowing the FPC to influence other cortical areas with which it is connected. It suggests that when a non-reward or unsatisfactory reward signal reaches the FPC, it disrupts the FPC’s stabilizing influence on other prefrontal regions, halting the continuation of ongoing behavior. This disruption triggers a switch mechanism that facilitates the exploration of alternative actions. Our results could be interpreted in this framework considering the reward representation in the Delay epoch, during which medium and large reward trials were represented separately from small reward trials. In the first case, this signal could inform about the fact that the best strategy is to continue selecting the same target in the last runs, where the percentage of switched trials is lower and consequently the performance is higher. Conversely, in small reward trials, it may represent a signal to continue with an exploratory strategy, leading to a greater number of switch trials in the latest runs, with the effect of slowing down the learning curve and worsening performance. This tendency to explore the alternative target in small reward trials is not related to a higher number of errors in this condition as it occurs even after a correct choice. The presence of an explore-exploit signal is supported by the coding by FPC neurons, in each scene, of the stay/switch decision with respect to the choice made for the same scene in the previous run. This signal was independent from the encoding of the reward magnitude and appeared later in time with respect to it, straddling the end of the Delay epoch and the choice of the target, probably elicited by the go signal instructing the animals to start moving. 

Future studies should investigate the monitoring role of the frontal pole further. It is possible that different experimental paradigms may highlight how the FPC monitors other aspects of the actions performed, beyond its spatial position and value, such as the difficulty, the effort required, or other perceptual characteristics of a chosen stimulus. This role as a ‘monitoring center’ is in line with its pattern of connectivity that shows dense reciprocal connections with the other prefrontal areas, making it particularly well suited to receive input of a different nature, and with the results of the few but consistent neurophysiological studies showing an involvement of neurons in this area in encoding different aspects of an action when it is performed. The monitoring function could also extend to other sensory modalities, e.g. auditory, thanks to the connections the FPC has with areas outside the prefrontal cortex, such as the superior temporal sulcus and the superior temporal gyrus in the anterior temporal lobe^94,95^. These results suggest that it is possible to think of the FPC as an information integration center, where in a two-step process information is first stored and then integrated and reprocessed to influence the activity of other cortical areas.

The present study has limitations at different levels. First, the task’s structure, despite enhancing rapid learning can lead to some confounds. During the first run, the behavior can only be explorative, each target was not yet associated with its corresponding outcome. Successively, monkeys can exploit the information learned in the first run, independently of whether they succeeded to select the correct target or not, since the task was deterministic and each scene presented a binary choice. It does not allow to discriminate whether the switch strategy adopted in small reward trials reflects a lack of learning or a monkey’s voluntary choice. Future investigations should introduce unknown objects when the associations are learned to give the monkeys the possibility to actively choose whether to explore new options or not. Second, the distinction between a satisfactory and an insufficient reward is somewhat arbitrary and is based on the monkeys’ prior experience. In a previous study using the same task^37^, the behavioral results emphasized the importance of the context in which learning takes place and how it is reinforced. The value of the reward is contextualized according to the available reward amounts and influences the learning curves accordingly. In the present study, monkeys had previous experience with a similar task^34^ but with only one amount of reward available for all scenes, corresponding to the medium reward in this version. This might explain why the learning rate was slower only in the small reward condition and elicited a switch strategy after the first run, even if this means changing a previously correct answer linked, however, to a small reward. Further studies will be needed to understand how the FPC is able to dynamically adjust the explore-exploit signal based on the subjective reward value.

## Methods

### Subjects

Two male monkeys (*Macaca mulatta;* Monkey 1: age 7 years, weight ~ 8 kg; Monkey 2: age 15 years, weight ~ 17 kg) were part of this study. Monkey 1 was obtained from R C Hartelust BV, located in 5049 Tilburg, Netherlands. Monkey 2 was obtained from Istituto di Biologia Cellulare e Neurobiologia, CNR, located in 00123 Santa Maria di Galeria, Rome, Italy. The animal care staff, the researchers involved in the study and a veterinarian monitored the general health and welfare of both monkeys daily. Animal care, housing, and experimental procedures conformed to the European (Directive 210/63/EU) and Italian (DD.LL. 116/92 and 26/14) laws on the use of non-human primates in scientific research. The research protocol was approved by the Italian Health Ministry (Central Direction for the Veterinary Service, authorization n°1211/2015-PR).

### Behavioral task

#### Task: general structure

The monkeys were trained to perform a variant of the object-in-place task, a task designed to investigate fast-learning, called object-in-place reward task (OIPR). During the neural recordings, the monkeys faced a touch screen monitor (3 M MicroTouch M1700SS 17" LCD touch monitor, 1280 × 1024 resolution) while sitting on a primate chair with their heads fixed. Two non-commercial software packages, CORTEX (NIMH, Bethesda, Maryland, United States of America, for Monkey 1) and MonkeyLogic (NIMH, Bethesda, Maryland, USA, for Monkey 2), were used to display images on the monitor, control a peristaltic pump delivering the reward, and record the behavioral responses of the monkeys. X and Y coordinates of the eye during task execution were monitored and recorded in all sessions for Monkey 2 through the ViewPoint Eye Tracker system (Arrington Research, Scottsdale, USA). Both monkeys were familiar with the experimental procedures such as the use of the monitor touch screen or the reward delivery system due to their previous experiences in several behavioral tasks and neural recording procedures^33,34,36,37,96^. During the OIPR task (described as performed in the current study in Ferrucci et al.^37^), the monkeys observed 5 different scenes, each one composed of a colored background with different geometrical shapes randomly selected and two target objects (Fig. 1A, left), located in two out of ten possible spatial positions (Fig. 1A, right). One object was the correct one, which if selected led to receiving a reward, while the other object was the incorrect one, which led to receiving no reward. For each position of the correct target, the incorrect target was presented in one of the remaining 6 positions excluding the three closest to the correct target to ensure adequate distance and discriminability between the positions of the two targets. For each correct choice, the monkey could receive three different reward sizes: a small reward, a medium reward, or a large reward, respectively ~ 0.15 ml, 0.3 ml and 0.6 ml of juice. Before the reward was delivered, different types of visual feedback (Fig. 1B) were presented around the chosen object, indicating the correctness of the choice (white squares or red hexagons) and the size of the reward (1 to 3 shapes). Similarly, for error trials the feedback indicated an incorrect choice (white circles and blue hexagons) and the size of the missed reward (1 to 3 shapes). The association between the scenes and the reward size was balanced to have all three sizes represented within the five scenes and never three times the same reward size. Once the monkey completed the 5 different scenes, the same scenes were presented again in the same order for a total amount of 6 repetitions or runs (Fig. 1D). The association between each scene and a specific reward size was the same in all the six runs. One block was defined as the completion of 30 trials (5 scenes × 6 runs), and once one block was completed, a new block started with 5 completely new scenes never seen before, each of them again associated with a specific reward size for all the 6 runs.

#### Task: trial structure

A specific scene was presented during a single trial (Fig. 1C). A trial started with the presentation of a white circular target at the center of the touch screen (CT, central target). The monkeys were required to touch and hold the touch on the white CT for a fixed period of time (Holding CT: 500 or 800 ms) in order to make the scene appear on the screen. After an additional period of time in which the monkeys still had to hold the touch on the white CT (Delay epoch: 800 or 1200 ms), the CT disappeared, representing the go-signal for choosing one of the two objects in the scene. After a holding period on the chosen object (Holding PT epoch: 400 or 600 ms), the visual feedback was presented around the object and remained on the screen for a variable period (Feedback epoch: 400 or 600 ms). After the Feedback epoch, the reward was delivered in correct but not in error trials. Any trial abort during any epochs preceding the feedback presentation led to the repetition of the same trial from the beginning.

### Surgery and data collection

High-density microelectrode chronic systems were bilaterally implanted in the two monkeys to record extracellular activity in the dorsal part of the FPC, also defined as lateral FPC (CerePort Utah Array, Blackrock Microsystems, Salt Lake City, UT, USA; a 96-channel array in each hemisphere for Monkey 1; a 48-channel array in each hemisphere for Monkey 2), as already described in previous works^33,34^. We used a fully automated algorithm to perform spike-sorting (MountainSort V4^97^). The dataset used in this study was composed of 234 single neurons recorded during 14 sessions (i.e., recording days), for a total of 94 blocks of 30 trials (44 blocks for Monkey 1 along 4 different sessions; 50 blocks for Monkey 2 along 10 different sessions). For Monkey 1, all the sessions were recorded only from the right hemisphere. For Monkey 2, 8 out of 10 sessions were recorded simultaneously in both hemispheres while the remaining 2 sessions were recorded only from the left hemisphere. From Monkey 1, 91 neurons were recorded. From Monkey 2, 143 neurons (54 from the right hemisphere and 89 from the left hemisphere) were recorded.

### Analysis of behavior

We calculated the percentage of correct trials for each run and reward size across all blocks separately for Monkey 1 and Monkey 2 (Fig. 2A). To evaluate whether the percentages of correct trials were significantly different from what would be expected by chance we performed a one-sided binomial test (p < 0.05) comparing the proportion of correct trials for each run/reward size with the probability expected by chance (0.5, since only two objects were presented in each scene). We calculated the reaction times (Fig. 2B,F), the time from the go-signal, i.e. the disappearance of the white CT to the beginning of movements toward the chosen objects (indicated by the detach from the screen) in all completed trials. Statistical differences between the reaction times distributions were assessed with a two-sample Kolmogorov–Smirnov test (p < 0.05). We investigated whether the performance differed between trials with different reward sizes comparing the proportion of correct responses in each run for each possible comparison (Small vs Medium, Small vs Large, Medium vs Large, Fig. 2C,G). Statistical differences between proportions of correct trials were assessed with a chi-square test (p < 0.05). We calculated the proportion of switch trials for each reward size in Runs 2 to 6. Switch trials are defined as those scenes in which the monkeys chose a different target than the one chosen in the same scene at the previous run. Statistical differences between proportions of switch trials in each run for each possible comparison (Small vs Medium, Small vs Large, Medium vs Large, Fig. 2D,H) were assessed with a chi-square test (p < 0.05). Finally, we calculated the proportion of switch trials for each reward size when the previous trial was a correct choice. Statistical differences between proportions of switch trials for each possible comparison (Small vs Medium, Small vs Large, Medium vs Large, Fig. 2E,I) were assessed with a chi-square test (p < 0.05). As a control, we repeated the analyses shown in Fig. 2A,C,D,E,G,H,I, using an alternative method. Instead of calculating proportions by collapsing all trials across recording days, we computed the measurements separately for each recording day and then averaged the results (Supp. Figure 2).

### Population decoding

We used a decoding procedure to evaluate the contribution of the whole population of neurons in the discrimination of the reward size in two different epochs: the Delay epoch and the Feedback epoch (Fig. 3A). We trained a linear support vector machine classifier (SVM, kernel = linear, C = 1) with a k-fold cross-validation procedure using neurons with at least 60 trials per condition (small, medium and large reward, n = 160: 91 for Monkey 1 and 69 for Monkey 2). We created a pseudo population randomly selecting 60 trials per condition for each neuron; then, each neuron activity was binned (250 ms with moving steps of 25 ms) in the two epochs of interest around the appearance of the scene (Delay epoch) and around the appearance of the visual feedback (Feedback epoch). Firing rate was normalized with a z-score transformation and the SVM classifier was trained on the activity of all neurons in k-1 trials and tested on the activity of all neurons in the remaining trials, repeating the train test split k times to use all trials as test trials once. This procedure was repeated n times (50) performing different resamples of trials to test different pseudo populations. The classification accuracy obtained in each time bin was the average classification accuracy across the n resamples. To test the significance of the average classification accuracy, we repeated the same procedure 20 times for each time bin randomly shuffling the condition labels (50 resamples × 20 shuffling of the labels for each bin). Then we collapsed the average across resamples for each shuffle and each bin to obtain a null model distribution of 1260 (20 shuffles × 63 bin) shuffled averages. For each bin we standardized the real classification accuracy value relative to the null distribution by computing its z-score. The significance of each observed value was determined by computing the proportion of values in the null distribution that were less than or equal to the observed z-score. Values with a p-value less or equal to 0.05 divided by the number of bins x the number of shuffles (Bonferroni corrected, α = 3.96 × 10–5) were considered significant. We used the same procedure to investigate whether the same population was able to discriminate the action (Fig. 4C) performed in the same scene between different runs (160 neurons, 25 trials per condition for each neuron). Trials were divided into two categories, excluding trials from the first run and without distinguishing by reward size: ‘stay’ trials, those scenes in which the same object chosen at the previous run was chosen, and ‘switch’ trials, those trials in which a different object was chosen from the previous run.

For each neuron, we performed a linear model analysis (*fitlm* function in Matlab), taking as regressors, the reaction-movement time (from go signal to touch peripheral target, different in each trial) and the reward size (categorical variable, small / medium / large) and their interaction. We then applied an ANOVA on the model (*anova* function on Matlab) to assess whether the neural activity was significantly modulated by each variable (Supp. Table 1) and to measure the percentage of variance explained by these variables.

To investigate how the information about reward size was encoded across runs within a block in the two epochs of interest (Fig. 3B) we performed the same decoding procedure with some changes. For each time bin, the SVM classifier was trained and tested in a moving window of 10 trials in each block. In this way, we selected the first 10 trials of each block (trials from 1 to 10; this resulted in a selection of 100 trials in an example recording session with 10 blocks) and performed the decoding procedure as described above. We then moved the window in sliding steps of 1 trial, selecting trials from 2 to 11 in each block, and so on, until we performed the decoding procedure in 21 different windows across the block (last window: from trial 20 to trial 30). The average classification accuracy was plotted as a heatmap where the first window is represented by the first row from the bottom. Statistical significance was again assessed by shuffling the labels and repeating the whole procedure. We run the decoding procedure on the same population of neurons (n = 160), with each neuron recorded for at least 20 trials per condition in every trial window.

### Disentangling action coding from reward coding

As revealed by the behavioral analyses, in the runs following the first one there is a strong correlation between reward and action labels, so medium and high reward trials are predominantly stay trials and conversely low reward trials are predominantly switch trials. To rule out the possibility that action coding represents a reflection of reward size coding we performed again a decoding procedure with a multi-variable balanced sampling of trials^98^. This procedure breaks the correlation between the variables by ensuring that training and test trials of each variable are sampled balancing the values of the correlated variable. We grouped medium and large reward trials together and performed again the decoding procedure for both the reward size (small vs medium-large) and the action (stay vs switch) in the 200 ms bin where we found the highest classification accuracy for the action (from 800 to 1000 ms after the appearance of the scene). We run the decoding in the selected bin on the same population of neurons used for all the previous analysis (n = 160), using a 70–30 split for training and test and a tenfold cross-validation procedure. Only neurons with at least 10 trials for each combination of variables were used. Statistical significance was assessed comparing the results with a null model obtained with 25 shuffling of the labels.

### Projections onto the discriminant

Similarities between reward size conditions in the neural activity of the whole population (Fig. 4A) were assessed by investigating the distance in the state space using a procedure similar to that adopted in previous work (a more detailed description can be found in Nougaret et al. 2024). Briefly, to represent the similarity in the neural state space, we constructed a linear discriminant between the two extreme values of reward size, small and large, and afterwards we projected the neural activity of the three reward size conditions onto the discriminant. We followed this procedure: for each neuron (n = 160, the same populations used for decoding), we binned the activity into 250 ms bins with 25 ms overlap in the two epochs of interest around the appearance of the scene (Delay epoch) and around the appearance of the visual feedback (Feedback epoch). Similarly, to the decoding procedure, for each bin we created pseudo populations of neurons by randomly sampling 60 trials per condition for each neuron, and we used a k-fold cross-validation procedure to divide data in training and test splits. We created two matrices: a train matrix of shape n x c, where n is the number of neurons (160) and c is the number of conditions (3), and each entry is the average activity in that bin across k-1 trials for that neuron in that condition; test matrix of shape n x c, where each entry is the average activity in the remaining test trial for that neuron in that condition. Both matrices were normalized for each neuron using the mean and the standard deviation calculated between training trials of the small and large reward conditions. The linear discriminant was calculated by subtracting the population vector of activities of training trials of the small reward condition from the one obtained from the training trials of the large reward condition, and then the projections for each condition were obtained with the dot product between the discriminant and the population vector of activities in the test trial of the three conditions. This procedure was repeated k times to use all the trials as test trials once and the remaining trials to generate the discriminant and to obtain average projections across the k repetitions. Then the procedure started again with a different pseudo population obtained randomly resampling 60 different trials for each neuron and repeated 100 times to obtain a distribution of projections for each condition. Significant differences across each pair of projections at each time bin were assessed by computing the proportion of the overlapping area between the probability density functions of 2 distributions, defined by an overlapping index^99,100^. Overlapping index indicating an overlap higher than 5% was considered not significant.

## Data Availability

The datasets generated during and analyzed during the current study is available at https://gin.g-node.org/lorenzo_ferrucci/FP_OIP3R.git.

## Code availability

Data were processed using MATLAB 2019a (https://www.mathworks.com). Decoding analyses were performed by using scripts of the Scikit-learn SVC package (scikit-learn.org). The multi-variable balanced sampling of trials decoding procedure to disentangle the correlated variables was performed using the Decodanda Python package (https://github.com/lposani/decodanda). Custom Matlab code to calculate the discriminant projections is available at https://gin.g-node.org/lorenzo_ferrucci/FP_OIP3R.git.
